# Preclinical mouse model to monitor live Muc5b-producing conjunctival goblet cell density under pharmacological treatments

**DOI:** 10.1371/journal.pone.0174764

**Published:** 2017-03-29

**Authors:** Céline Portal, Valérie Gouyer, Frédéric Gottrand, Jean-Luc Desseyn

**Affiliations:** LIRIC UMR 995, Univ. Lille, Inserm, CHU Lille, Lille, France; Save Sight Institute, AUSTRALIA

## Abstract

**Purpose:**

Modification of mucous cell density and gel-forming mucin production are established hallmarks of mucosal diseases. Our aim was to develop and validate a mouse model to study live goblet cell density in pathological situations and under pharmacological treatments.

**Methods:**

We created a reporter mouse for the gel-forming mucin gene *Muc5b*. Muc5b-positive goblet cells were studied in the eye conjunctiva by immunohistochemistry and probe-based confocal laser endomicroscopy (pCLE) in living mice. Dry eye syndrome (DES) model was induced by topical application of benzalkonium chloride (BAK) and recombinant interleukine (rIL) 13 was administered to reverse the goblet cell loss in the DES model.

**Results:**

Almost 50% of the total of conjunctival goblet cells are Muc5b^+^ in unchallenged mice. The decrease density of Muc5b^+^ conjunctival goblet cell population in the DES model reflects the whole conjunctival goblet cell loss. Ten days of BAK in one eye followed by 4 days without any treatment induced a −18.3% decrease in conjunctival goblet cell density. A four days of rIL13 application in the DES model restored the normal goblet cell density.

**Conclusion:**

Muc5b is a biological marker of DES mouse models. We bring the proof of concept that our model is unique and allows a better understanding of the mechanisms that regulate gel-forming mucin production/secretion and mucous cell differentiation in the conjunctiva of living mice and can be used to test treatment compounds in mucosal disease models.

## Introduction

Mucus gels are an essential defensive barrier on most secretory epithelia. The major functions of mucus gels include lubrication and hydration of epithelial tissues. The properties of mucus are mainly driven by their gel-forming mucin content. Gel-forming mucins are large heavily *O*-glycosylated macromolecules produced by specialized secretory cells before being excreted as multimers into the lumen to form mucus gel when in contact with water. The five genes *MUC2*, *MUC5AC*, *MUC5B*, *MUC6*, and *MUC19* encoding the five gel-forming mucins in humans are conserved in mice [1,2] and are designated *Muc2*, *Muc5ac*, *Muc5b*, *Muc6*, and *Muc19*. Gel-forming mucin production is regulated at two main levels: at the gene expression level (gene regulation) and by cell differentiation from undifferentiated cells into mucous cells. Abnormal or dysregulation of gel-forming mucin expression and abnormal mucous cell density are hallmarks of many mucosal diseases and mucosal damage.

Dry eye syndrome (DES) is a common and multifactorial disorder affecting ~10–20% of the population worldwide [3]. In this disease, the conjunctival goblet cell (CGC) density is inversely proportional to the disease severity [4]. Treatments capable to improve symptoms and to restore the homeostasis of the ocular surface are needed and, today, there are only few pharmaceutical treatments available [4,5]. New drugs or treatments need to be validated using *in vitro* techniques but also relevant whole animal models with good molecular markers [6]. Mice became an invaluable tool for discovering therapeutic targets due to the similarities between human and mouse eyes coupled to the ability to manipulate easily the mouse genome. Among the five gel-forming mucins, mouse Muc5b and Muc5ac are both produced by the CGC [7,8] and may represent good molecular markers of CGC density. MUC5B is secreted as long homo-polymers [9]. Its gene has been fully sequenced showing an uncommonly long 10.7-kb central exon coding for regions enriched in Ser and Thr that carry the high number of oligosaccharide chains characteristic of all mucins [10]. The *MUC5B* gene is conserved in the mouse genome [1], allowing the generation of a Muc5b-deficient mouse strain showing that Muc5b is important for mucociliary clearance in the lung during bacterial infection [11]. We report here the creation of a genetically modified mouse strain in which the gel-forming mucin Muc5b was tagged by homologous recombination with a green fluorescent protein (GFP) sequence. We show that gel-forming mucin production and epithelial differentiation of the conjunctiva into Muc5b mucin-secreting goblet cells can be easily monitored in living mice using probe-based confocal laser endomicroscopy (pCLE) [12] in unchallenged mice and in a DES model using topical application of benzalkonium chloride (BAK), a common preservative used in ophthalmic agents. We next bring a proof of concept that our transgenic model is very valuable to test drugs in DES. To this aim, we first showed that the Muc5b-positive (Muc5b^+^) CGC decrease in the DES model reflects the total CGC loss imaged in living mice. Next, we tested if the restoration of a normal CGC density can be measured using pCLE in living mice. For this purpose we used IL13 topical application as IL13 mediates goblet cell hyperplasia and *MUC5B* upregulation in normal human bronchial epithelial (NHBE) cells [13], induces MUC5B mRNA expression and MUC5B production in primary human bronchial epithelial cells [14], increases the proportion of secretory cells in human nasal epithelial cells in primary culture [15] and has been shown to directly increase goblet cell formation producing MUC5B in primary human airway basal cell culture in Matrigel [16]. Furthermore, IL13 has been reported to stimulate mucin production in cultured conjunctival epithelium [17].

## Materials and methods

### Mice

Mice were housed in a pathogen-free facility, and all of the experimental protocols were approved by the Animal Care Committee of the region Nord—Pas de Calais (protocol 1606–2015090217056239). The animal care and all procedures were in accordance with the French Guide for the Care and Use of Laboratory Animals and with the ARVO Statement for the Use of Animals in Ophthalmic and Vision Research. Heterozygous (Muc5b^gfp/+^) and homozygous (Muc5b^gfp/gfp^) Muc5b-GFP mice were used throughout this study using their wild-type (WT) littermates as a negative control for fluorescence activity.

### Transgenic mouse

**Fig 1:**
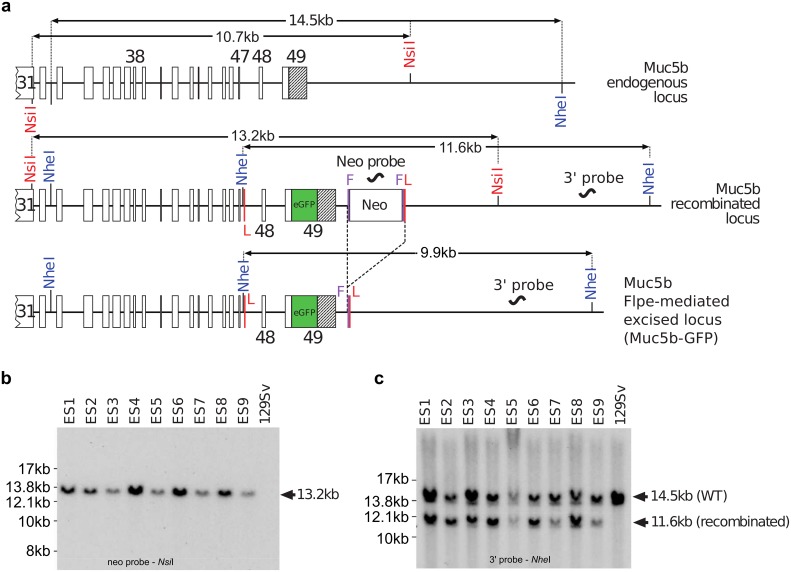
Gene targeting and analysis of targeted integrants. **(a)** Map of Muc5b recombined and Muc5b KI loci, and after Flpe-mediated excision of the positive selectable neomycin (Neo) marker. The FRT (F, vertical purple lines) and loxP (L, vertical red lines) sites are indicated. The loxP sites were not used in this study. The *Nsi*I and *Nhe*I restriction sites used for Southern blot experiments are shown. The *Nhe*I restriction within intron 47 does not exist in the wild-type (WT) allele. Exons are indicated by rectangles and few of them are numbered. The unique 3’ untranslated region (UTR) is shown in black and belongs to the 49^th^ and last exon. **(b)** Southern blot analysis of homologous recombination in nine embryonic stem (ES) cell clones. Genomic DNA of the ES cell clones was compared with WT DNA (129SvPas ES cells). Correct targeting by homologous recombination is indicated by a 13.2-kb band obtained with a *Neo* probe on *Nsi*I digested DNA whereas no band is visible for the WT as expected. **(c)** A 11.6-kb and 14.5-kb band obtained with the external 3’ probe on *Nhe*I-digested genomic DNA for the recombined locus and the WT allele, respectively.

**Fig 2:**
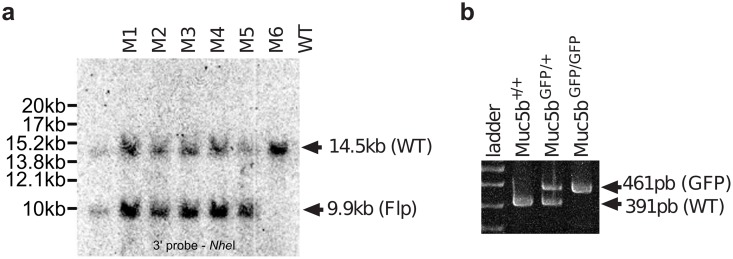
Generation of a Muc5b-GFP KI mouse. **(a)** Southern blot analysis of six (M1 to 6) heterozygous Flpe-excised KI mice. The genomic DNA of tested mice was compared to the DNA of a wild-type (WT) mouse. *Nhe*I-digested DNA was blotted onto a nylon membrane and hybridized with the external 3’ probe showing a 14.5-kb band for the WT allele and a 9.9-kb band for the Neo-excised allele (KI Muc5b-GFP) in agreement with the restriction map (see Fig 1a). **(b)** Example of tail-DNA genotyping by PCR.

The gene targeting strategy used to generate Muc5b-GFP knock-in (KI) mice was designed in collaboration with genOway (Lyon, France), where the unique Stop codon of *Muc5b* was replaced by a synthetic nucleic sequence encoding a Gly-Ser-Ile-Ala-Thr peptide followed by the monomeric enhanced GFP sequence [18]. The enhanced GFP sequence was in frame with the last amino acid of Muc5b, which is located in the last exon (exon 49) of the gene. In addition, a 2.2-kb genomic region comprising the two last exons 48 and 49 was flanked by loxP sites to create a knockout model that was not used in the experiments described here. A *Neo* selective cassette flanked by two FRT sites was introduced downstream from the last exon of *Muc5b* (Fig 1a). The short arm and long arm homologous sequences were obtained by PCR from 129Sv/Pas embryonic stem cell DNA using proof reading thermostable Taq polymerase “Accuprime Taq DNA Polymerase High Fidelity” (Invitrogen). PCR products were subcloned into the pCR4-TOPO vector (Invitrogen) by TA-cloning. The sequences obtained from PCR amplification of 129SvPas genomic DNA were first aligned with each other to identify putative mutations introduced by the PCR amplification step. The short arm homologous sequence was 2197-bp long, containing exon 49 downstream sequence, and was amplified using the following primers: 5’-GCACTGCTCAAACACCAAGAGGCTG-3’ (upstream) and 5’-GGAGGTCAGAGGACAACTTTTGGAGTCA-3’ (downstream). The long arm was amplified by a two-step procedure. A 3135-bp sized fragment containing exons 38 to 47 and neighboring intronic sequence was amplified using the primers 5’-CCCGCTTCCTTGGTTCTTCTGAACC-3’ (upstream) and 5’-GCTTTTGGTGGGAACTGGATGGAGC-3’ (downstream) and then cloned to generate the distal part of the long homologous arm of the targeting vector. A 2184-bp sized fragment containing exons 48 and 49 and neighboring intronic sequences was amplified using the two primers 5’-AATACAAGTTTCTCCCACGGGATGGC-3’ (upstream) and 5’-AAAGCACCCTTCTCCACGTGTCTGC-3’ (downstream) and then cloned to generate the proximal part of the long homologous arm. At least three independent subclones for each amplification were fully sequenced and at least one clone for each genomic fragment was shown to contain no mutation within the region used for the targeting vector construction. The isolated 129Sv/Pas sequences were aligned with the C57BL/6 sequence available in public databases showing no polymorphism between the two genetic backgrounds C57BL/6 and 129Sv/Pas. A full construct was generated with a synthetic monomeric enhanced GFP sequence, the flipped-*Neo* cassette and loxP sites. The quality of the resulting final targeting vector was controlled by sequencing of the coding exons, the junctions between the homologous arms and the selection cassette, the junctions between the homologous arms and the plasmid backbone and the reporter cassette. Gene targeting into 129Sv/Pas embryonic stem cells was performed according to GenOway's electroporation procedures (i.e. 10^8^ embryonic stem cells in the presence of 100 μg of the 13.6-kb *Pme*I linearized plasmid, 260 Volts, 500 μF). Positive selection was started 48 h after electroporation, by the addition of 200 μg/mL of G418 (150 μg/mL of active component; Life Technologies, Inc.). A total of 262 resistant clones was isolated and amplified in 96-well plates. Duplicates of 96-well plates were made. The set of plates containing embryonic stem cell clones amplified on gelatin was genotyped by both PCR and Southern blot analysis as follows. Isolated clones were screened by PCR using the two primers 5’-GTGAGACGTGCTACTTCCATTTGTCAC G-3’ (upstream) and 5’-GTTGTAAGTTCAGGCAAAGGATCAAGACG-3’ (downstream, *Neo* cassette) to test for homologous recombination at the 3' end of the Muc5b locus. Thirty positive clones displayed an amplified product of the expected size of 3051-bp. The recombined clones identified by PCR were further verified by Southern blot analysis of *Nsi*I-digested embryonic stem cell DNA using an internal 5’ probe located within the *Neo* cassette of the targeting vector (Fig 1b). A homologous recombination event at the 3’end of the targeting vector was controlled by Southern blot using DNA digested with *Nhe*I and hybridization with an external 3’ probe located downstream of the homologous sequence of the targeting vector (Fig 1c). Three recombined embryonic stem cell clones were injected into C57BL/6J blastocysts that were then re-implanted into OF1 pseudo-pregnant females. Male chimeras were obtained and mated with C57BL/6 Flpe deleter female mice to achieve germ-line transmission with excision of the neomycin selection cassette in order to generate heterozygous mice carrying the *Neo*-excised KI allele (Muc5b^gfp/+^). Heterozygous *Neo*-excised Muc5b^gfp/+^ KI mice referred to thereafter and in the main manuscript as the Muc5b-GFP mouse line, were further verified by Southern blot analysis using the external 3’ probe and genomic DNA digested with *Nhe*I (Fig 2a). Mouse genotypes were then routinely determined by PCR using the two primers 5’-GTCAGGCATCTCATGCTCACAAAAGC-3’ and 5’-AGGATGTAGGGTCCTAGCACCAATGTAGC-3’ (Fig 2b).

### Mouse genotyping

Mouse genotypes were determined by PCR analysis of tail DNA using the two primers 5’-GTCAGGCATCTCATGCTCACAAAAGC-3’ (upstream) and 5’-AGGATGTAGGGTCCTAGCACCAATGTAGC-3’ (downstream) leading to an amplification product of 461- and 391-bp for the excised KI and WT allele, respectively.

### Tissue collection, stereomicroscopy, histology, and immunohistochemistry

Mice were killed and tissues were removed and rinsed in phosphate-buffered saline (PBS) (Gibco BRL, France). For stereomicroscopic observations, pictures of eyelids were taken using a M205 stereomicroscope (Leica) equipped with a color DFC450c camera (Leica). Eyeball with eyelids was fixed in 4% paraformaldehyde for 20 h and paraffin embedded. Transversal 4 μm tissue sections located in the middle of the eyeball were cut. Sections were stained with either periodic acid-Schiff (PAS) or alcian blue (AB)–PAS as described previously [19,20]. Immunohistochemical studies using the 45M1 antibody directed against MUC5AC was performed as described previously [19,20]. The primary polyclonal anti-GFP antibody (Ab290) was purchased from Abcam, France. Nuclei (blue) were counterstained with Hoechst 33258. Histological and immunofluorescence analyses were performed on a Leica DM4000B. To determine the number of Muc5b^+^ and Muc5ac^+^ CGC relative to total CGC visualized by histology (AB—PAS staining), serial sections were used from eight non-challenged transgenic mice. To confirm the decrease in total CGC, Muc5b^+^ and Muc5ac^+^ CGC in the DES model by histology and immunohistochemistry, one histological section and two serial immunohistochemical sections (anti-GFP antibody and 45M1) of each eye (one receiving PBS, the other BAK) from ten transgenic mice were first used. The number of goblet cells in the superior and inferior conjunctiva was measured in 1 section from each eye from 10 mice that received both PBS and BAK and also by averaging the data from three nonconsecutive cross-section slides from each eye that were at least 240 μm apart.

### *In vivo* fibered confocal laser microscopy

Mice were anesthetized using ketamine (100 mg/kg) and xylazine (10 mg/kg). Live GFP activity was recorded by pCLE using a Cellvizio apparatus and an ultra-thin flexible fibered microprobe ProFlex S-0650 (Mauna Kea Technologies, Paris, France) as described previously [21]. Movies were acquired by sweeping a large area of the conjunctiva under the eyelid with the pCLE probe. Recording started at the temporal side and the complete round of the eye was done in 25 s. Extracted frames (one every five frames) from movies were analyzed using Fiji [22] to determine, blinded to the treatment, the integrated density per count and the number of GFP^+^ CGCc. CGC counting was performed on the entire area of each frame, corresponding to the total acquisition field of the probe. Each analyze by Fiji was performed using between 60 and 80 frames.

### Dry eye model and pharmacological treatment

The DES model was induced as described by Liu *et al*. by topical application of 5 μL of 0.2% BAK (B6295; Sigma Aldrich, France) or PBS as a control twice a day for 10 days [23]. Topical BAK treatment induces an ocular surface injury with a decrease of CGC, a component of disease mechanism that leads to dry eye. Preliminary experiments showed that the decrease of CGC density after 10 days of BAK treatment is still observed by pCLE after 4 days without any treatment following the 10-day time course of topical BAK application, leaving a 4-day window time period to test a drug treatment. To stimulate CGC differentiation, topical administration of IL13 was administrated. Based on published data on topical application of IL28A [24] and preliminary data obtained in our laboratory on rIL13 in mice with DES, we used five ng of rIL13 (5 μL; SRP4166; Sigma Aldrich, France) that were administered by topical application twice a day for four days.

### Quantitative real-time RT-PCR

Eyelids and eyeball were dissected together from 20 mice. Eyeball was then removed before freezing the eyelid. Total RNA extraction was performed on the full conjunctiva (bulbar, fornical and palpebral; two conjunctiva from mice that received the same treatment/extraction) using the innuPREP RNA Mini Kit (Analytik Jena, Germany). RNA were reverse transcribed to synthesize cDNA using 0.2 U of MMLV Reverse Transcriptase (Promega, USA) and random hexamers according to the manufacturer's instructions. Amplifications using 18S rRNA as an internal control (TaqMan Ribosomal RNA Control Reagents, Applied Biosystems, USA) were performed as previously described [25]. Oligonucleotides and probes used to measure *Muc5b* and *Muc5ac* gene expression have been published previously [25]. Amplifications were performed in triplicate with a 7500 Applied System (Applied Biosystems, USA). For each sample, the ratio of amplification is calculated as 2^-(Ct_mean_ target gene − Ct_mean_ 18S).

### Statistical analysis

Beanplots were generated using BoxPlotR [26]. Nonparametric Wilcoxon—Mann—Whitney test, paired permutation test and Spearman's correlation test were performed using StatXact6.0 (Cytel Studio, Cambridge, MA). The paired *t*-test was performed using R freeware. A *P* value <0.05 was considered significant.

## Results

### Generation of Muc5b-GFP KI mice

**Fig 3:**
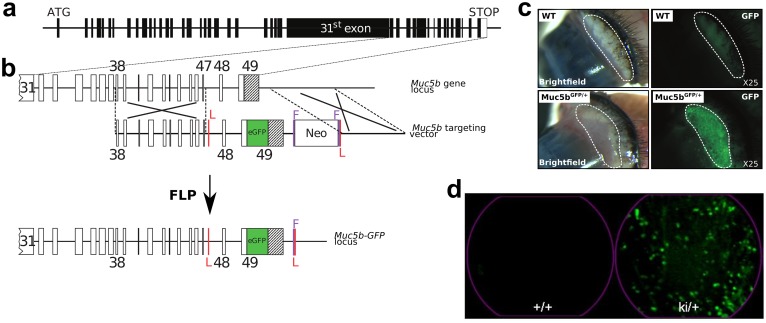
Strategy for creating Muc5b-GFP knock-in (KI) mice and fluorescence stereo- and endomicroscopy. **(a)** *Muc5b* gene structure. Human and mouse *Muc5b* are composed of 49 exons indicated by rectangles (black, coding sequence and white, 3’UTR) and introns by lines. The large 31^st^ exon carries the sequences encoding peptides enriched in Ser+Thr+Pro. The initiation (ATG) and STOP codons are indicated. **(b)** Strategy for replacing the unique STOP codon of the endogenous mouse *Muc5b* gene with an enhanced GFP sequence (in green). A Gly-Ser-Ile-Ala-Thr linker is placed between the last amino acid of Muc5b and a synthetic sequence coding for a monomeric enhanced GFP sequence. Exons are indicated by rectangles and few of them are numbered. The unique 3’UTR region in black belongs to the 49^th^ and last exon. The targeting vector contains 3.2-kb of upstream (exons 38–47) and 2.2-kb of downstream sequences from the mouse Muc5b locus. LoxP sites (red vertical lines), which played no role in these experiments, flank the two last exons of *Muc5b*. A *Neo* positive selectable marker for embryonic stem cell integration flanked by two FRT sites (purple vertical lines) was inserted downstream of the Muc5b locus. The Muc5b-GFP line was obtained by crossing mice with a Flpe recombinase-expressing mouse in order to delete the neomycin cassette used for selection in embryonic stem cells. **(c)** Representative examples of pictures in bright-field mode and under GFP excitation by stereomicroscopy of fresh excised conjunctivas from wild-type (WT) and transgenic (Muc5b^GFP/+^) mice. **(d)** Extracted frames from representative movies from conjunctivas acquired by pCLE (see S1 Movie) from a WT (+/+) and a transgenic mouse (ki/+).

Heterozygous and homozygous Muc5b—GFP KI mice (Muc5b^ki/+^) were successfully produced by homologous replacement of the endogenous 3’ end of the mouse *Muc5b* gene where the unique Stop codon was replaced in frame by a synthetic peptide, GSIAT, fused to the monomeric enhanced GFP (Figs 1–3b). The positive *Neo* cassette for embryonic stem cell selection was removed by mating chimeric mice with a Flpe-deleter mouse strain. Correct events of homologous recombinations were checked by Southern blot experiments on DNA extracted from embryonic stem cells and transgenic mice (Figs 1 and 2). All transgenic pups grew up healthy and adult homozygous and heterozygous transgenic mice and their progeny were viable and fertile.

### Muc5b expression in fresh eye conjunctiva

We looked for GFP fluorescent activity using epifluorescence stereomicroscopy in eye. An intense signal was observed in eye conjunctiva while no GFP activity was detected in control WT mice (Fig 3c). We next investigated GFP activity in the conjunctiva using pCLE. Illustrative extracted frames are shown in Fig 3d. GFP activity appeared as fluorescent spots reflecting GFP^+^ goblet cells or clusters of goblet cells (S1 Movie). Autofluorescence coming from hairs and possibly from cellulose debris was also sometimes observed in WT and transgenic mice. Collectively, these data demonstrate that GFP is easily detectable in eye conjunctiva in anesthetized mice by pCLE.

### Fluorescent activity in eye conjunctiva to monitor DES

**Fig 4:**
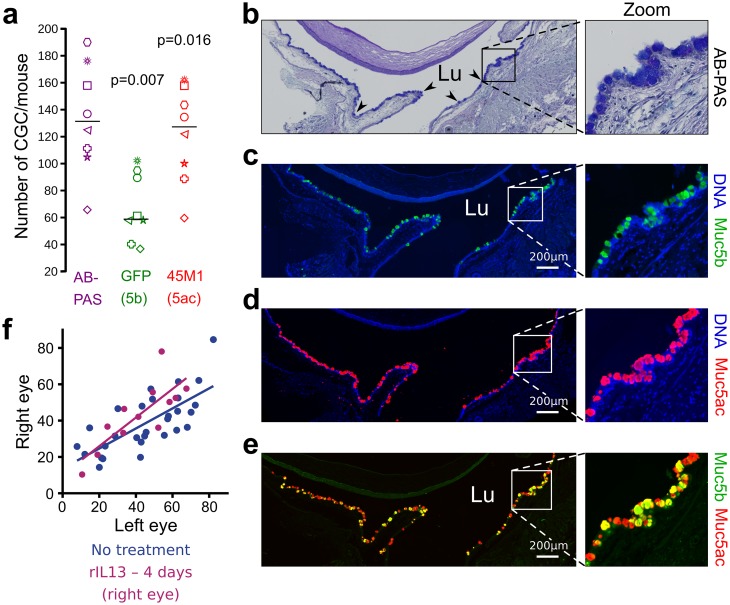
Similar GFP activity in eye conjunctiva after topical application of rIL13. **(a)** The number of CGC, GFP^+^ (Muc5b) and 45M1^+^ (Muc5ac) CGC were counted using serial sections from untreated mice, one for AB—PAS staining and the other for immunofluorescence staining using an anti-GFP antibody and the anti-Muc5ac antibody (45M1). N = 8 transgenic mice; two-sided Permutation test, *P* = 0.007 and *P* = 0.016, respectively. Each symbol represents one mouse. Sample median is shown as horizontal bars **(b-e)** Representative AB—PAS histological section and immunohistochemical serial sections stained with anti-GFP (Muc5b, green) and anti-Muc5ac (red) from a Muc5b^GFP/+^ mouse conjunctiva showing GFP^+^ and Muc5ac^+^ CGC. The four arrowheads illustrate several goblet cells of the conjunctiva. Lu, lumen; scale bar, 200 μm. **(f)** Number of GFP^+^ CGCc determined by pCLE analysis in each eye of 12 mice (purple) which received rIL13 in the right eye and PBS in the left for 4 days in comparison to 29 mice (blue) without any treatment. Statistical analysis was performed using paired *t*–test.

We next tested whether the GFP reporter gene is valuable to test drugs in mouse models of DES. The number of GFP^+^ and Muc5ac^+^ (45M1 antibody) CGC in the conjunctiva represents 49 ± 9.8% and 91.1± 9.1% of total number of CGC, respectively (Fig 4a–4e). CGC co-localization of Muc5ac and Muc5b by immunofluorescence supported that some CGC produce Muc5ac alone, some other Muc5b alone and a last subset of CGC produces the two gel-forming mucins. The Spearman correlation coefficient (r_s_) between the AB—PAS and GFP^+^ cell number showed a strong positive correlation (r_s_ = 0.91, two-sided *P* = 0.003) supporting a direct link between the total CGC and Muc5b^+^ cell density. We then verified that the density of GFP^+^ CGC clusters (CGCc) recorded by pCLE was similar in the two eyes (Fig 4f). As a drug, we tested whether rIL13 stimulates CGC differentiation in our model of DES. Without DES, 4 days of rIL13 topical application had no effect on the GFP^+^ CGCc density (Fig 4f).

**Fig 5:**
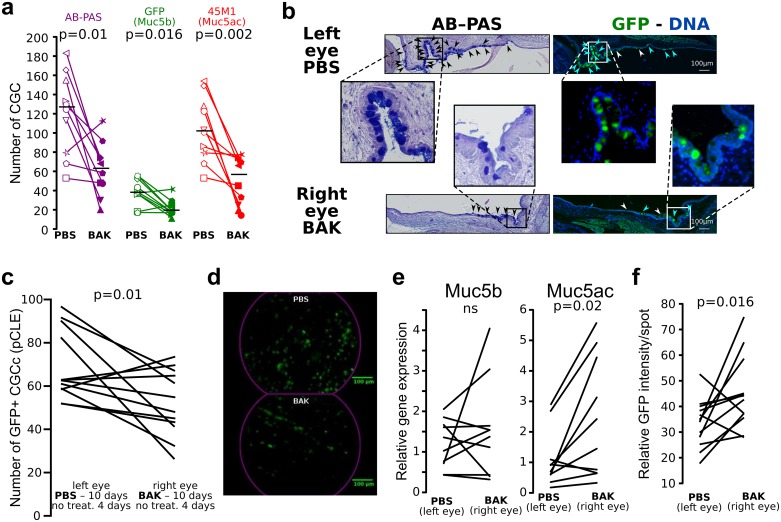
GFP activity is decreased in DES. **(a)** Scatterplots show that CGC number determined on histological sections (AB—PAS coloration), and GFP^+^ (Muc5b) and Muc5ac^+^ CGC number determined by immunofluorescence staining (anti-GFP and anti-Muc5ac antibody) decreased (n = 10 mice, 1 section per mouse, one symbol/mouse) after 10 days of BAK application (filled-in symbols) in one eye (PBS in the other, white-filled symbols) followed by 4 days without any treatment. Horizontal lines indicate median results. **(b)** Histological and immunofluorescence staining. AB—PAS and serial immunohistochemical section of the left eye, which received PBS, and paired-right eye which received BAK show a decrease in the number of conjunctival goblet cells producing Muc5b (blue arrows) and non-Muc5b producing (white arrows) cells. Black arrows indicate goblet cells. Green signal, anti-GFP antibody. Nuclei (blue) were counterstained with Hoechst 33258. Scale bar, 200 μm. **(c)** Scatterplot showing that the number of GFP^+^ CGCc determined by pCLE decreased significantly (n = 12 mice; *P* = 0.01) after 10 days of BAK application in one eye (PBS in the other) followed by 4 days without any treatment. **(d)** Illustrative extracted frames of pCLE recorded movies from a conjunctiva after 10 days of PBS application in comparison to 10 days of BAK application followed by 4 days without any treatment. Scale bar, 100 μm. **(e)** Scatterplots showing the expression levels of the two gel-forming mucins *Muc5b* and *Muc5ac* determined by RT-qPCR (TaqMan). No significant (ns) change in gene expression levels of *Muc5b* was found while *Muc5ac* was significantly up-regulated after 10 days of topical BAK application (n = 10 extracted RNA/treatment; *P* = 0.02). **(f)** Scatterplot showing the integrated fluorescence density per GFP^+^ CGCc determined by Fiji showing an up-production of Muc5b/CGCc after 10 days of BAK treatment (n = 12 mice; *P* = 0.016). Statistical significance was assessed using one-sided (a) or two-sided permutation test (a, e, f). ns, *P*>0.05.

To establish the DES responsiveness of the reporter gel-forming mucin *in vivo*, we subjected transgenic mice to topical applications of BAK in one eye for 10 days. Mice were sacrificed for analysis 4 days after the last BAK application. AB—PAS CGC, GFP^+^ and Muc5ac^+^ CGC were counted on one section from 10 mice which received PBS in the left eye and BAK in the right. The ratio of GFP^+^/AB—PAS and Muc5ac^+^/AB-PAS CGC were 0.30 and 0.8 for the PBS-treated eyes, respectively, and 0.33 and 0.73 for the right BAK-treated eyes, respectively. The BAK treatment induced a 48%, 46% and 52% decrease in AB—PAS, Muc5b (GFP^+^) and Muc5ac (45M1^+^) CGC, respectively (Fig 5a and 5b). To secure these data, we also performed the same analysis using three independent sections from 7 mice (Table 1). This confirmed that Muc5b (GFP^+^) and Muc5ac (45M1^+^) CGC represent 45% and 89% of the total CGC number, respectively in the control eye (left eye) and 43% and 89% of the BAK-treated eye (right eye). Furthermore, BAK treatment induced a decrease of 54%, 57% and 55% of the total AB-PAS, Muc5b and Muc5ac positive CGC, respectively, highlighting a perfect correlation between the Muc5b^+^ CGC density and the AB-PAS^+^ CGC density in both the control and the stressed eye. Analysis of GFP^+^ CGCc by pCLE in 12 mice showed a −21.1% (± 26.9) decrease in GFP^+^ CGCc density compared to paired-eye PBS control (Fig 5c and 5d, S2 Movie). These data show that the decrease in CGCc density, a key feature in DES, can be easily monitored and quantified in living Muc5b-GFP transgenic mice. Furthermore, a 4-day window after the last BAK application can be used to test any treatment in the DES model.

**Table 1:** CGC counting (7 mice, 3 sections/mouse) in left (PBS) and right eye (BAK).

	PBS (left eye)					BAK (right eye)							
	AB-PAS	GFP		45M1		AB-PAS		GFP			45M1		
Mouse	Count^a^ ±SD^b^	Count^a^ ±SD^b^	%	Count^a^ ±SD^b^	%	Count^a^ ±SD^b^	vs PBS (%)^c^	Count^a^ ±SD^b^	%	vs PBS (%)^c^	Count^a^ ±SD^b^	%	vs PBS (%)^c^
1	131±2	68±3	52	107±3	81	52±5	61	18±5	34	74	44±6	85	59
2	280±4	98±7	35	245±6	87	115±4	59	33±5	28	67	97±2	84	60
3	141±1	47±3	33	111±4	79	78±1	44	23±3	29	51	65±8	83	42
4	118±7	53±3	45	104±3	88	48±7	59	20±2	42	62	44±1	90	58
5	102±3	56±1	54	98±3	96	35±6	66	25±1	71	56	35±6	100	65
6	89±2	40±1	45	86±2	96	61±9	32	26±2	42	36	48±8	79	44
7	95±4	48±8	50	93±3	98	38±7	60	21±1	56	56	38±7	100	59
**average**	**137±2**	**58±7**	**45**	**121±2**	**89**	61±5*	**54**	24±4*	**43**	**57**	53±9*	**89**	**55**

^a^ average;

^b^ standard deviation;

^c^ decrease relatively to the PBS eye control.

* *P* = 0.016 for AB-PAS, GFP (Muc5b) and 45M1 (Muc5ac), left eye *vs* right eye, 2-sided permutation test.

To begin to address if the decreased CGCc density may be accompanied by compensatory increases of gel-forming mucin expression, we assessed mRNA expression levels of *Muc5ac* and *Muc5b* in eye conjunctiva of mice which received BAK in one eye for ten day *vs*. PBS in the control eye. Interestingly, no modification was observed for *Muc5b* while a significant 2.2-fold increase was found for Muc5ac (Fig 5e, *P* non-significant and *P* = 0.02, respectively). Theses data suggested that the decrease of CGC following BAK treatment induces a compensatory upregulation of the two mucins which is more remarkable for *Muc5ac*. To confirmed that the decrease of GFP^+^ CGCc density is accompanied by a compensatory increase of Muc5b at the protein level, the integrated density of fluorescence per GFP^+^ CGCc was also determined. Consistent with a compensatory effect, BAK treatment induced a +44% increase of fluorescence per CGCc (Fig 5f, *P* = 0.016). Altogether, these data demonstrated a compensatory effect of the two main gel-forming mucins following CGC depletion.

**Fig 6:**
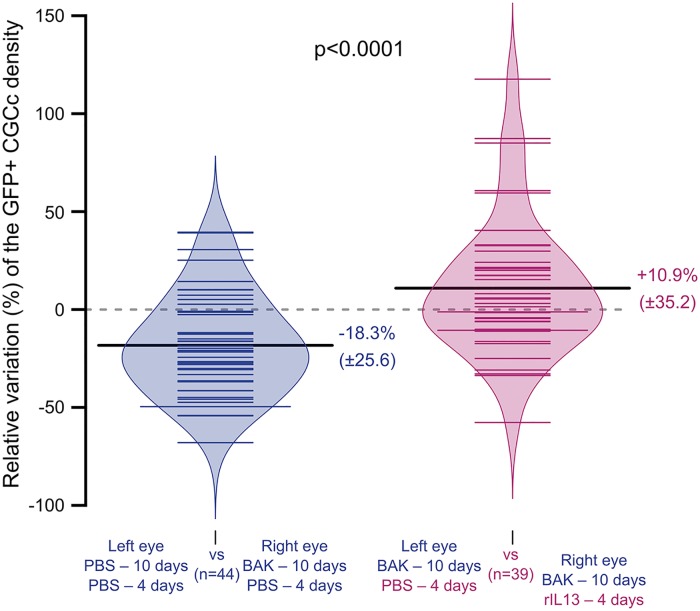
Depletion of CGC is restored by IL13. The beanplots show that daily topical rIL13 application in the DES model significantly restored (*P*<0.0001, one-sided Wilcoxon-Mann-Whitney test) the GFP^+^ CGCc density (n = 44, PBS-control mice (blue); n = 39, rIL13-treated mice (purple)). Mice from the control group received BAK for 10 days in one eye (PBS in the other) followed by 4 days of PBS in both eyes. Mice from the rIL13 group received BAK in both eyes for 10 days followed by rIL13 in one eye and PBS in the other for 4 days. The percentage of GFP^+^ CGCc in the treated eye vs. the non-treated eye was calculated for each mouse from pCLE movies. Sample median is shown as horizontal black bars.

To next show that rIL13 can stimulate CGC differentiation in DES, dry eyes induced by 10 days of topical BAK application were followed by 4 days of rIL13 topical application. A −18.3% (± 25.6) decrease in GFP^+^ CGCc density induced by BAK treatment was reversed by topical rIL13 application (increased of 28.6%) (Fig 6). This demonstrates that rIL13 reversed the decrease in CGCc density induced in DES.

## Discussion

The mouse has unquestionably become the vertebrate of choice to study development and gene function in mammals. It is also the most widely used preclinical model for determining the biology of diseases and testing drugs and new therapeutics. Gel-forming mucins are highly conserved between humans and mice in terms of gene number, structure and expression, suggesting that mice may represent a good model to study mucins and mucus physiology.

Conjunctival goblet cells are a major cell type in ocular mucosa [27]. Our study has shed some light on gel-forming mucin in the eye conjunctiva with unexpected data. Only one published study to our knowledge has revealed mouse Muc5b^+^ CGCc density [28]. The authors concluded that Muc5b is produced by only a few CGC while Muc5ac remained the major gel-forming mucin. However, this study was performed using a polyclonal antibody directed against a 19 amino acid peptide of human MUC5B [29] which shares only 47% similarity with its mouse Muc5b peptide counterpart. Our immunohistochemical and fresh tissue imaging unambiguously revealed that production of Muc5b in the eye is lower than that of Muc5ac. Yet, Muc5b production has been underestimated as it is produced by almost half of the total CGC. For this reason, it may represent a good marker for mouse models of DES. *MUC5B* gene has been reported to be expressed in human conjunctiva [30] but neither RNA nor protein has been found in the conjunctival epithelium and tear film [31].

Balance and distribution of epithelial cell types is required to maintain tissue homeostasis. An established hallmark of mucosal diseases is epithelial remodeling, leading to modification of mucous cell density and abnormal production of mucus [16]. Our Muc5b-GFP reporter mouse may help to monitor mucous cell density in other tissues of living mice where Muc5b has been shown to be produced, i.e. ear, nose, mouth, trachea and vagina [32]. Fluorescent gel-forming mucin represents a valuable tool to study diseases and treatments because mucous cells provide the basis for new treatments, and to test drugs involved in gel-forming mucin production. To date, amongst rodent gel-forming mucins, only mouse Muc19 has been tagged *in vivo* [33]. However, Muc19 expression is restricted to salivary mucous glands, submucosal glands of the trachea-larynx and to bulbourethral glands. Furthermore, the transgenic mouse is a reporter at the genetic level but not the peptide level because it was created by inserting a GFP peptide just downstream from the first 69 amino acids of mucin. In contrast, the Muc5b—GFP transgenic mouse was designed in order to tag the full mucin to study Muc5b expression under its native regulatory elements and to have a reporter mouse at the peptide level.

There is clearly a lack of knowledge concerning conjunctival goblet cell mucin synthesis and secretion, specially *in vivo* [27]. pCLE technology, which is a cost-effective technology, greatly empowers the study of live mucus in eye conjunctiva. This imaging technology is easy to set up but requires transgenic animals to specifically tag a gene product and access to a fiber microprobe confocal microscopy apparatus. Movies can be acquired without any specific training and the data are obtained very quickly. The decrease of CGC density after BAK treatment is only of 21.1% according to pCLE while immunohistochemistry analysis suggested a decrease of almost 50%. The main reason to explain this range of decrease is that CGC are organized in rodents in clusters interspersed between stratified squamous cells [31]. Consequently, it is difficult for Fiji software to make the difference between a unique spot (a cell) and a set of spots (a cluster of cells) although this matter may deserve to be further studied using a new specific plugin of the software. The DES we mimicked by BAK topical application may also explain the variability observed between mice as few mice responded weakly to the treatment. This may come from a too low BAK amount we administrated and from the administration method as mice were anesthetized for a short time period (less than 20 seconds) and tended to scratch out the drug. Nevertheless, pCLE enables longitudinal studies on anesthetized mice, reducing the number of mice required per experiment to better meet ethical guidelines. Movies acquired by pCLE support that the distribution of Muc5b^+^ CGC is heterogeneous. pCLE has advantage of obtaining a representative general picture of the GFP^+^ CGCc density of the whole conjunctiva in contrast to studies using histological sections because CGC are unevenly distributed [34]. Our data show that BAK treatment induces a decrease of CGC density but no decrease of Muc5b expression and an up-regulation of *Muc5ac* expression suggesting a compensatory effect of mucin production. This is supported by pCLE analysis which shows that the GFP intensity of the remaining goblet cells of BAK-treated eyes is higher compared to control eyes and in agreement with a recent report showing in primary murine culture model of CGC that IFN-γ increased expression of both *Muc2* and *Muc5ac* RNA levels but do not induce production of these mucin glycoproteins [35].

In conclusion, we report here that the mouse Muc5b^+^ CGC subpopulation reflects in normal situation and during an experimental DES model the whole CGC population. Our reporter Muc5b-GFP transgenic mouse allowed to follow the CGC density using live imaging to study DES and to monitor treatment efficiency in ocular diseases. The transgenic mouse should help to study other mucosal diseases where the Muc5b is produced.

## Supporting information

S1 Movie pCLE under the eyelid of Muc5b^+/+^, Muc5b^gfp/+^ and Muc5b^gfp/gfp^ mice. (AVI)

S2 Movie pCLE in the eye conjunctiva under pharmacological treatments (PBS or BAK) of Muc5b-GFP heterozygous mice. (AVI)
